# Supplementary material for: Extracellular Vesicles Derived from Osteogenic-Differentiated Human Bone Marrow-Derived Mesenchymal Cells Rescue Osteogenic Ability of Bone Marrow-Derived Mesenchymal Cells Impaired by Hypoxia
Source: Biomedicines. 2023 Oct 16;11(10):2804. doi: 10.3390/biomedicines11102804 (PMC10604262; doi:10.3390/biomedicines11102804)
Supplement: Supplementary file 1 [file biomedicines-11-02804-s001.zip › biomedicines-2569582-supplementary.pdf]

Supplementary Material

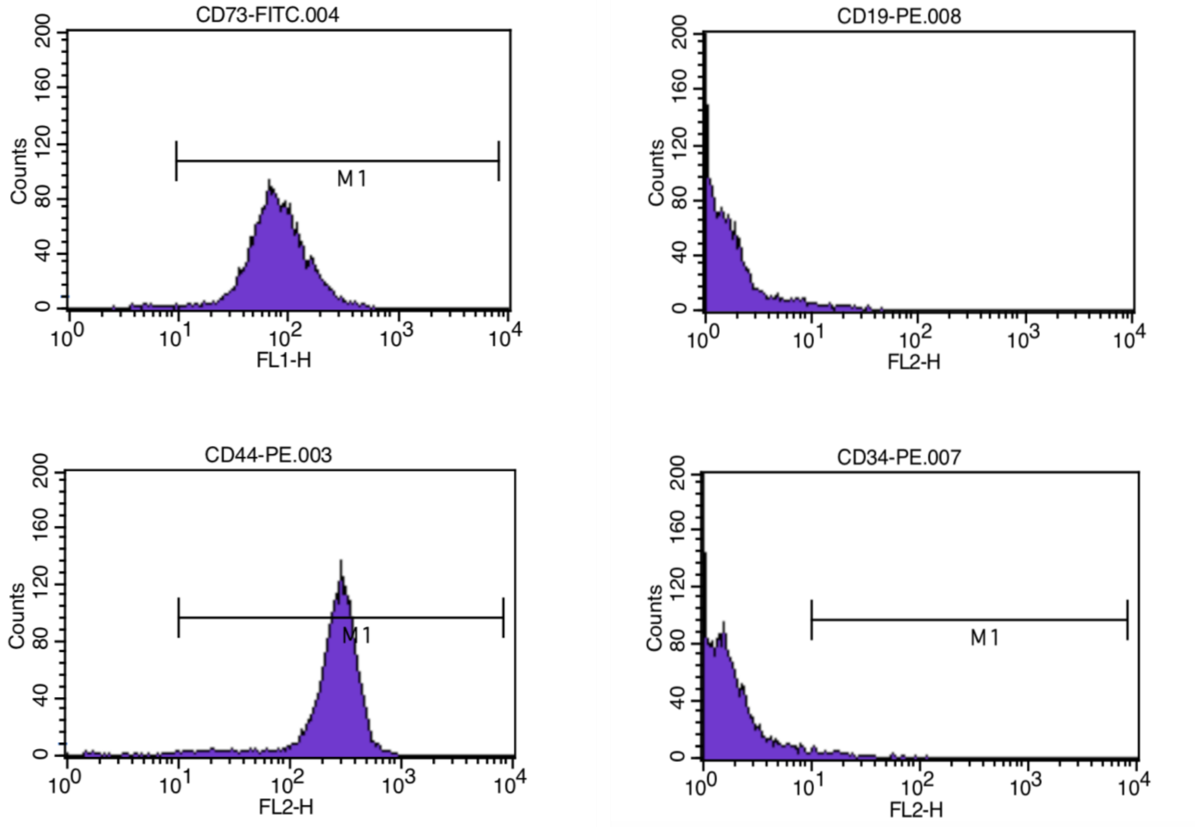

Figure S1. Flow cytometric analysis of hBMSC with specific antibodies against positive marker (CD44, CD73) and negative marker (CD19, CD34).

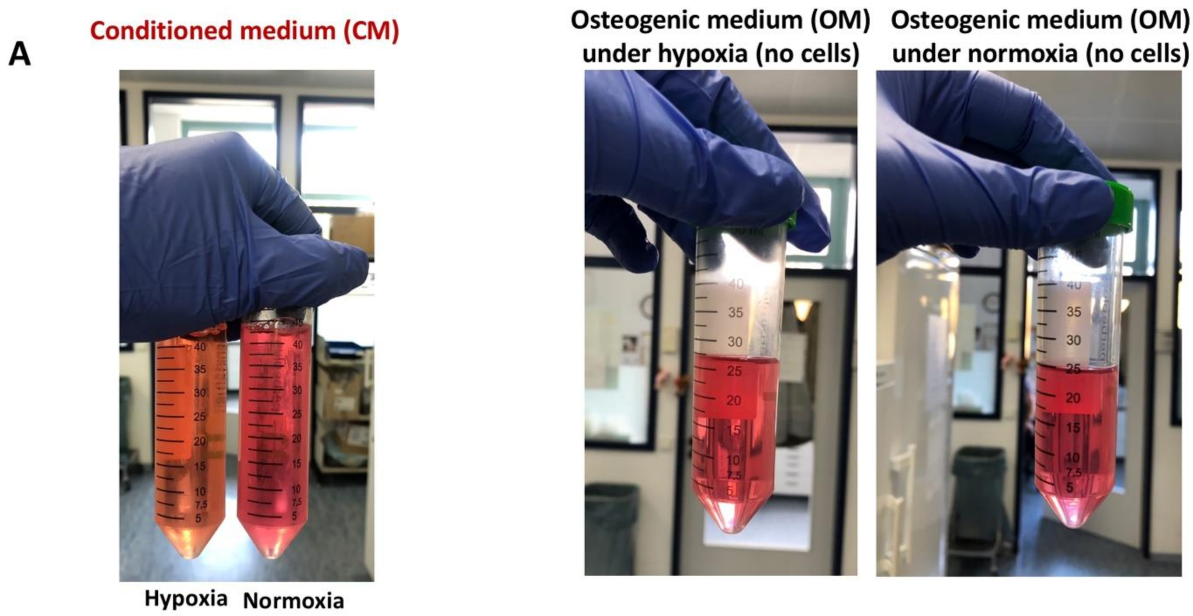

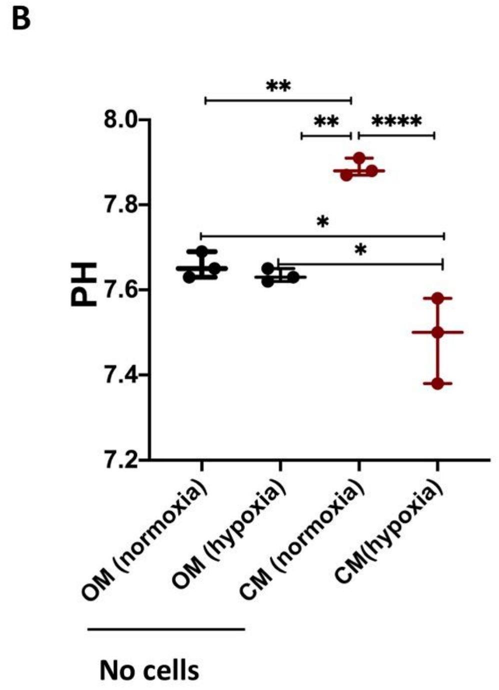

Figure S2. Conditioned medium from hypo-osteo EVs and norm-osteo EVs.

(A) Conditioned medium from hBMSCs undergoing osteogenic differentiation at 33-35 days under hypoxia and normoxia (CM). Osteogenic medium (OM, without subjecting to cell culture) was kept for two days under hypoxic and normoxic conditions.

(B) PH determination of the two conditioned media (CM) and the two osteogenic media (OM); n=3.

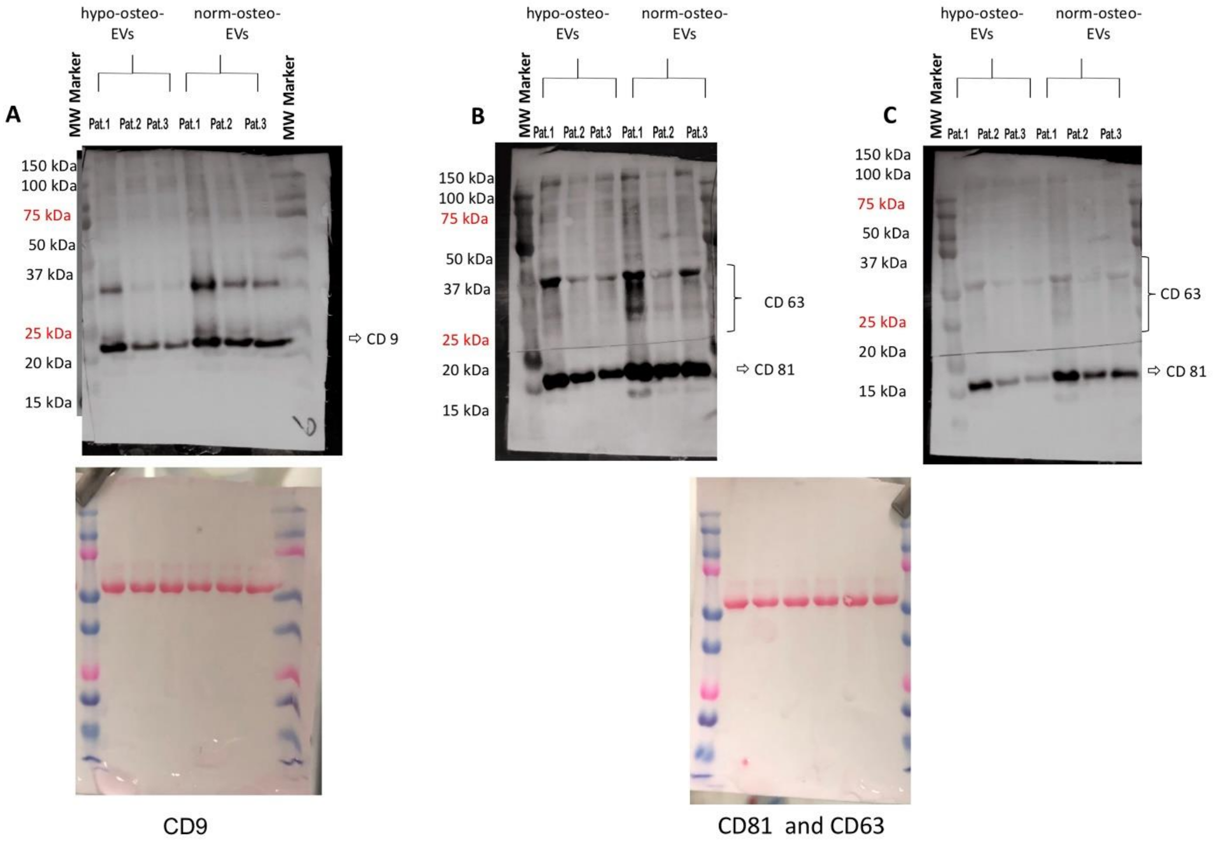

Figure S3. The uncropped western blot membranes of figure 2G and respective Ponceau Red stained loading control images below; n=3.

(A, C) Protein bands were detected using ECL detection reagents. （B）Protein bands were detected using SuperSignal West Femto Maximum Sensitivity Substrate.

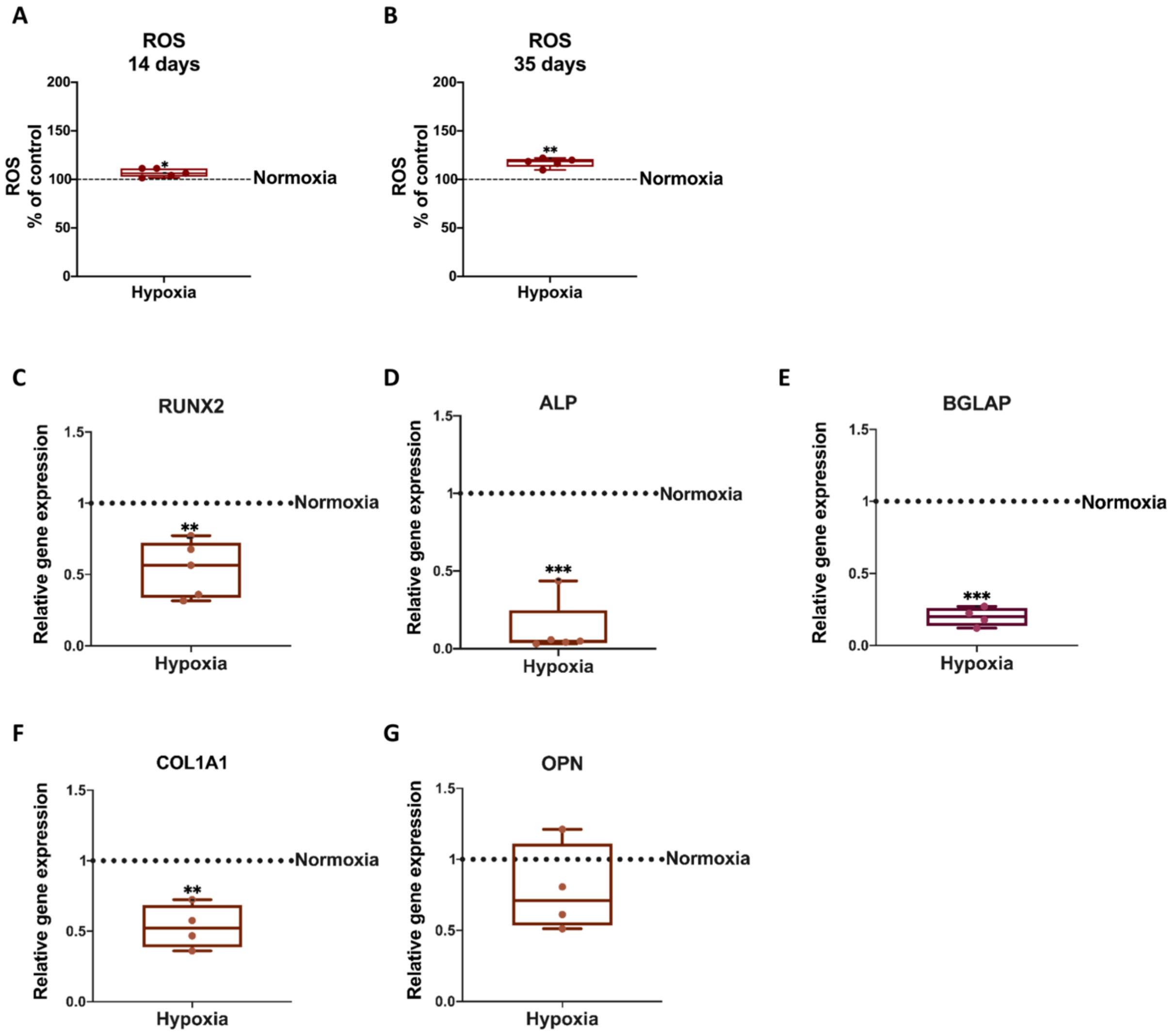

Figure S4. Evaluation of reactive oxygen species (ROS) secreted, and osteogenic marker gene expression in osteogenic differentiated hBMSCs under hypoxia and normoxia.

(A) Reactive oxygen species (ROS) secreted from hBMSCs after **14** days of osteogenic differentiation under hypoxia and normoxia; n=5.

(B) Reactive oxygen species (ROS) secreted from hBMSCs after **35** days of osteogenic differentiation under hypoxia or normoxia; n=5.

(C-G) Gene expression level of the osteogenic marker genes (OPN, BGLAP, ALP, RUNX2, and COL1A1) after 14 days of osteogenic differentiation of hBMSCs under hypoxia and normoxia; n=4-5.

Results were calculated as percentage to the control group (osteogenic differentiation of hBMSCs under normoxia, shown by the dotted line); *$p < 0.05$; **$p < 0.01$; ***$p < 0.001$;

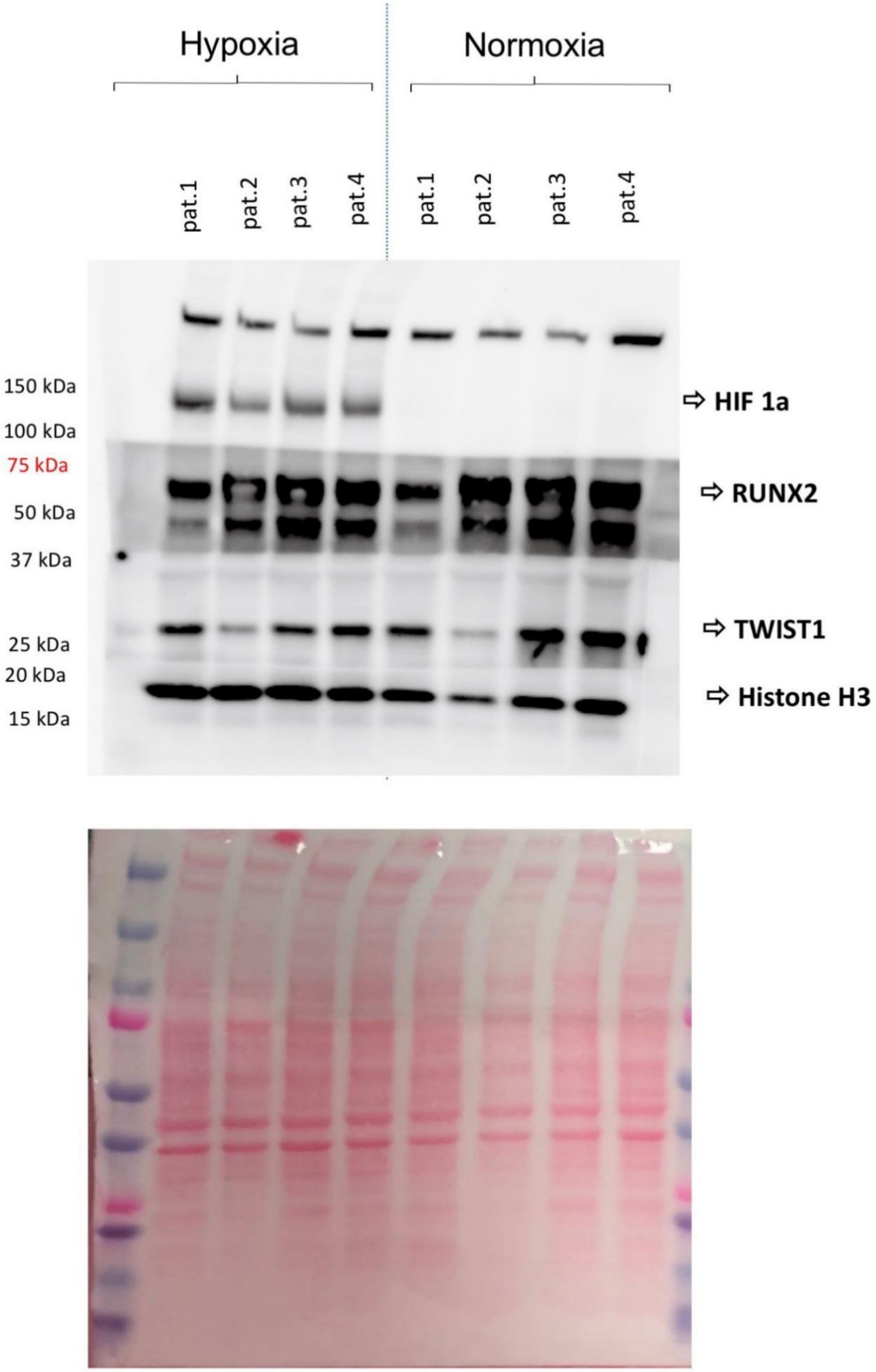

Figure S5. The uncropped western blot membrane of figure 4F and respective Ponceau Red stained loading control images below; n=4.

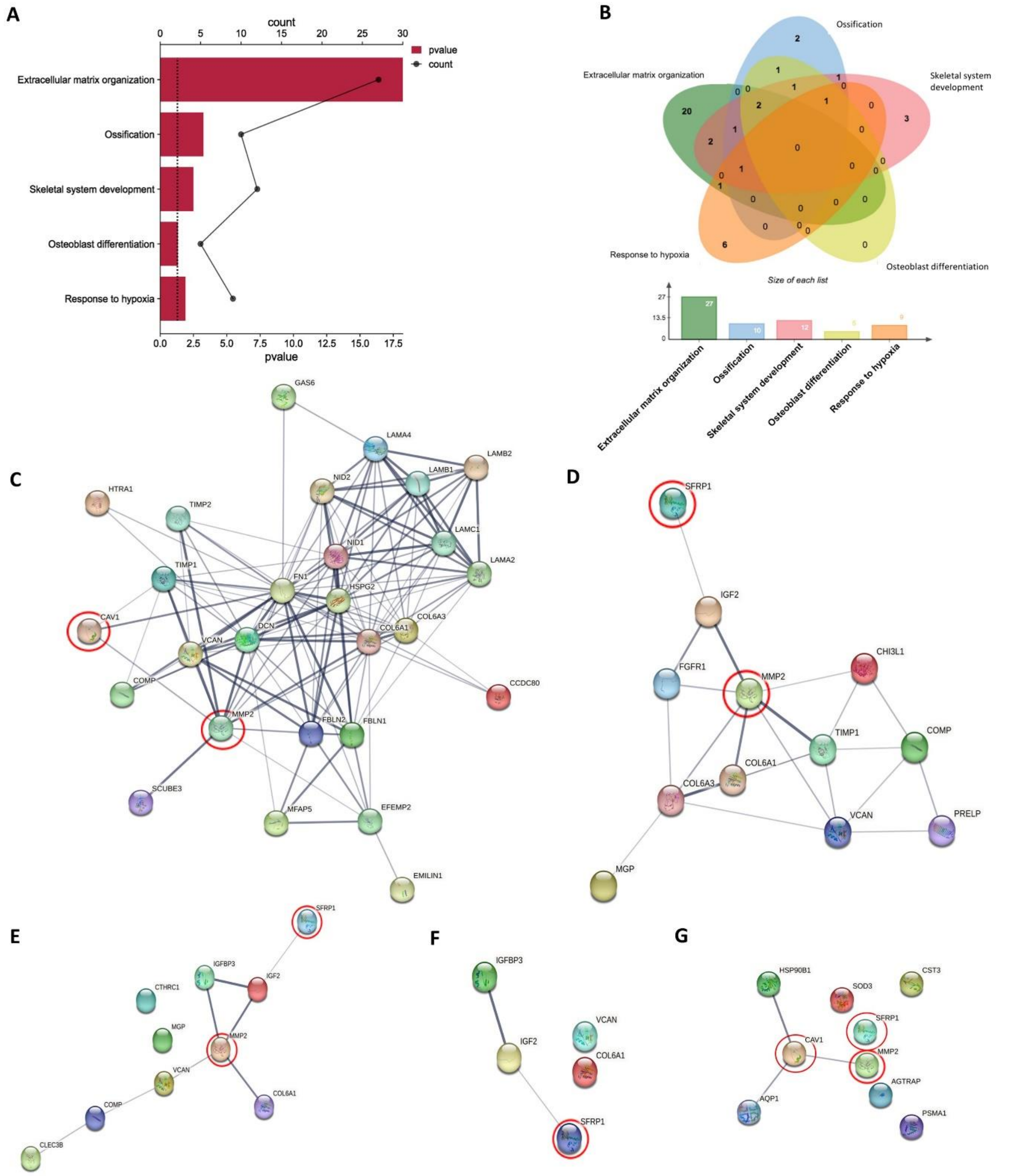

Figure S6. GO Enrichment analyses and protein-protein interaction (PPI) network analyses of norm-naïve EVs and norm-osteo EVs

(A) GO analysis for the "upregulated biological processes" related to hypoxia and osteogenesis in norm-osteo EVs compared with that in norm-naïve EVs.

(B) Venn diagram of 'response to hypoxia associated", 'ossification', 'skeletal system development', 'osteoblast differentiation' and 'extracellular matrix (ECM) organization'. PPI

networks of ‘ECM organization’ (C), ‘ossification’ (D), ‘skeletal system development’ (E), ‘osteoblast differentiation’ (F), and ‘response to hypoxia’ (G). The three proteins of ‘response to hypoxia’ (SFRP1, MMP2 and CAV1) in red circles are associated with ECM synthesis and osteogenesis; n=3.

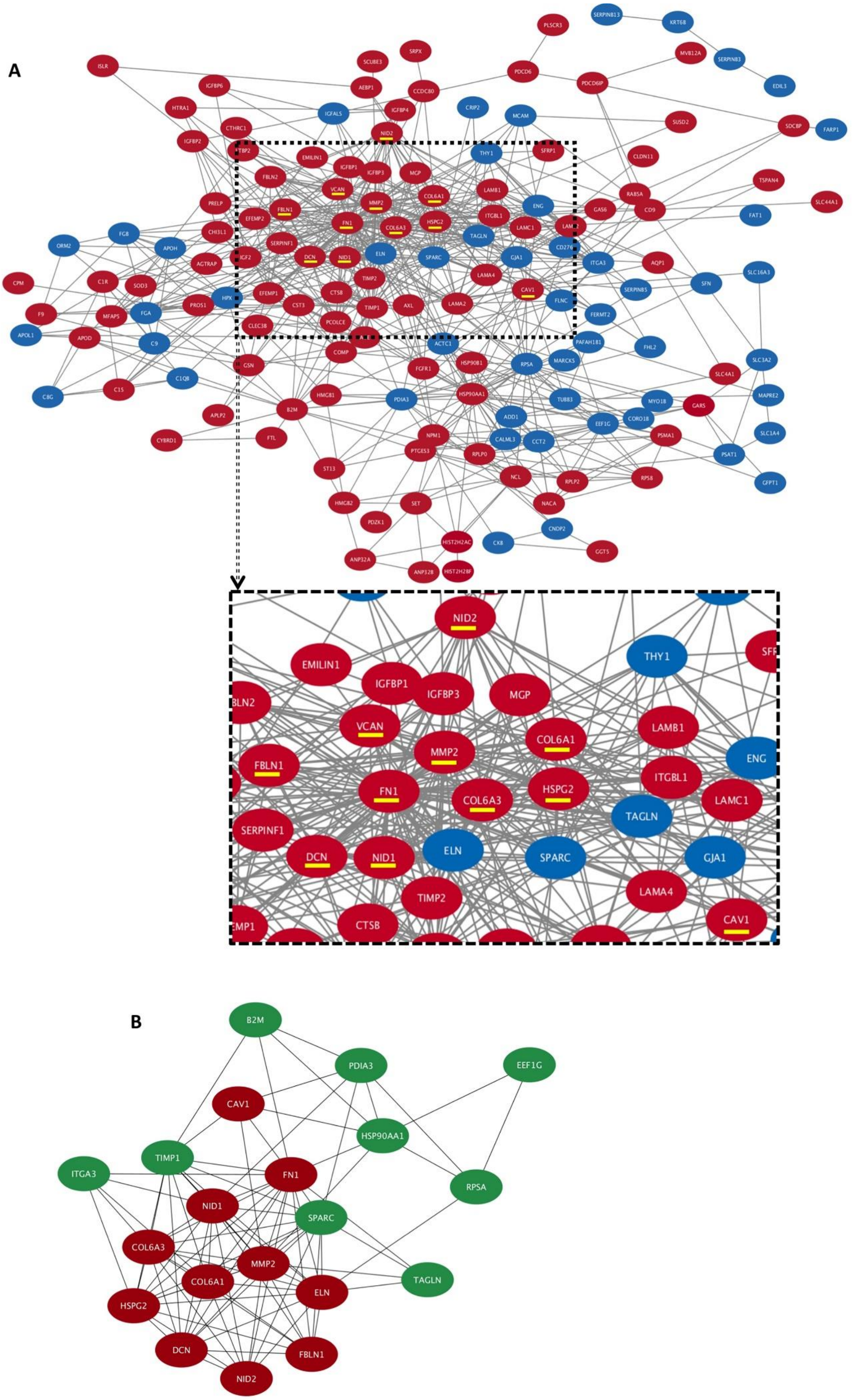
A
ISLR
IGFBP6
HTRA1
IGFBP2
CTHRC1
LTBP2
SCUBE3
AEBP1
SRPX
CCDC80
IGFBP4
IGFALS
CRIP2
MCAM
PLSCR3
PDCD6
PDCD6IP
MVB12A
SERPINB13
KRT6B
SERPINB3
EDIL3
SUSD2
SDCBP
FARP1
CLDN11
TSPAN4
SLC44A1
RAB5A
CD9
GAS6
FAT1
FGB
ORM2
APOH
PRELP
CHI3L1
AGTRAP
CPM
C1R
SOD3
F9
MFAP5
FGA
PROS1
HPX
APOL1
APOD
C9
C1QB
C8G
C1S
GSN
COMP
ACTC1
PAFAH1B1
FHL2
FERMT2
SERPINB5
SFN
SLC16A3
SLC3A2
SLC4A1
MAPRE2
MYO1B
GARS
CORO1B
EEF1G
PSMA1
SLC1A4
PSAT1
GFPT1
RPS8
RPLP2
NACA
NCL
CCT2
CALML3
ADD1
TUBB3
MARCKS
RPSA
HSP90B1
HSP90AA1
FGFR1
PDIA3
HMGB1
B2M
APLP2
CYBRD1
FTL
NPM1
PTGES3
RPLP0
ST13
HMGB2
SET
PDZK1
HIST2H2AC
CK8
CNDP2
GGT5
ANP32A
ANP32B
HIST2H2BF
NID2
EMILIN1
IGFBP1
IGFBP3
MGP
THY1
SFRP1
FBLN2
VCAN
MMP2
COL6A1
LAMB1
ENG
FBLN1
FN1
COL6A3
HSPG2
ITGBL1
LAMC1
EFEMP2
SERPINF1
DCN
NID1
ELN
SPARC
TAGLN
GJA1
CD276
ITGA3
AQP1
IGF2
TIMP2
LAMA4
CAV1
FLNC
EFEMP1
CTSB
CST3
TIMP1
AXL
LAMA2
CLEC3B
PCOLCE
B
B2M
PDIA3
EEF1G
CAV1
HSP90AA1
TIMP1
ITGA3
FN1
NID1
SPARC
RPSA
COL6A3
MMP2
COL6A1
ELN
TAGLN
HSPG2
DCN
FBLN1
NID2

Figure S7. PPI network of the distinct proteins and hub proteins identified in norm-osteo EVs compared with that in norm-naïve EVs .

(A) Interactions between upregulated and downregulated proteins in norm-osteo EVs compared with that in norm-naïve EVs; n=3; Red nodes indicate upregulated proteins, and blue nodes indicate downregulated proteins.

(B) The 20 most highly correlated hub proteins in PPI network. The red nodes indicate the 11 hub proteins related to hypoxia or osteogenesis. The 11 hub proteins are marked with a yellow line.

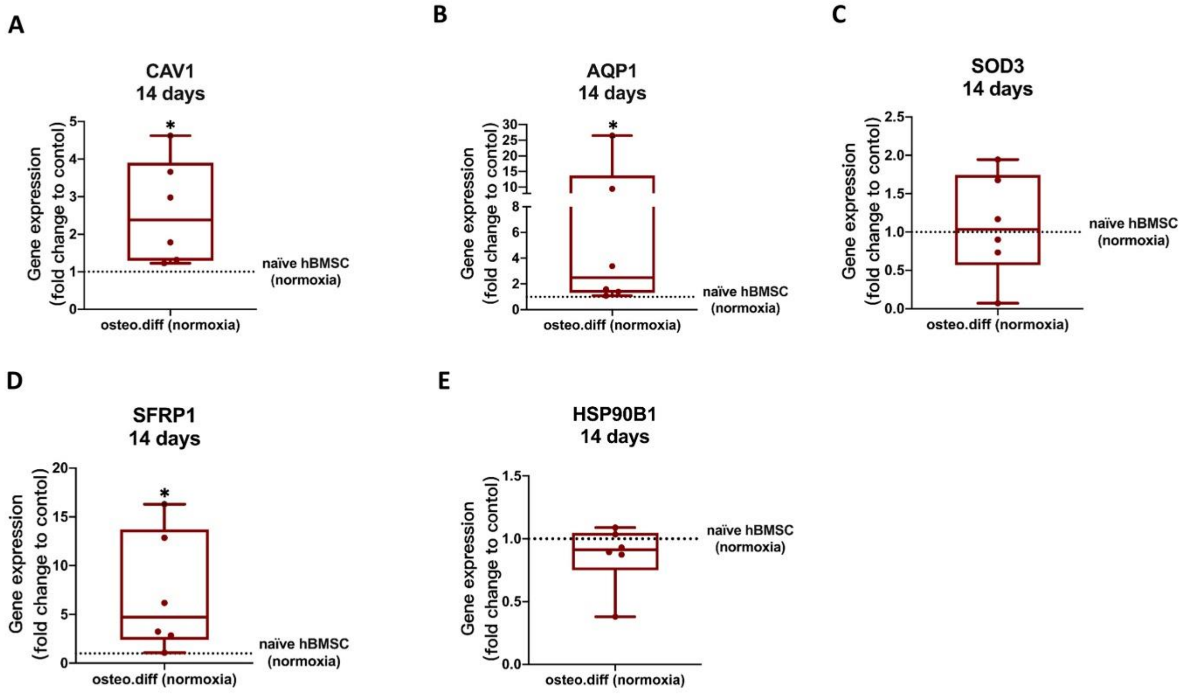

Figure S8. Validation of the proteomic data obtained from norm-naïve EVs and norm-osteo EVs by gene expression analysis in hBMSC.

(A-E) Expression level of the five anti-hypoxic genes were analysed in hBMSCs after **14 days** of osteogenic differentiation under normoxia and naïve hBMSCs under normoxia; n=6.

Tables

Table S1. Distinct proteins identified in hypo-osteo EVs and norm-osteo EVs (attached excel file).

Table S2. Primer sequences for qPCR.

Gene	Primer sequences (5’–3’)
GAPDH	Fwd, CTGACTTCAACAGCGACACC
	Rev, CCCTGTTGCTGTAGCCAAAT
TBP	Fwd, TTGTACCGCAGCTGCAAAAT
	Rev, TATATTCGGCGTTTCGGGCA
ALP	Fwd, CCTCCTCGGAAGACACTCTG
	Rev, CCTCCTCGGAAGACACTCTG
BGLAP	Fwd, GTGCAGAGTCCAGCAAAGGT
	Rev, TCAGCCAACTCGTCACAGTC
COL1A1	Fwd, ACGTCCTGGTGAAGTTGGTC
	Rev, ACCAGGGAAGCCTCTCTCTC
RUNX2	Fwd, CGGAATGCCTCTGCTGTTATG
	Rev, GCTTCTGTCTGTGCCTTCTG
OPN	Fwd, TGAAACGAGTCAGCTGGATG
	Rev, TGAAATTCATGGCTGTGGAA
SOD3	Fwd, ATGCTGGCGCTACTGTGTT
	Rev, CTCCGCCGAGTCAGAGTTG
CAV1	Fwd, GCGACCCTAAACACCTCAAC
	Rev, ATGCCGTCAAAACTGTGTGTC

SFRP1	Fwd, ACGTGGGCTACAAGAAGATGG
	Rev, CAGCGACACGGGTAGATGG
AQP1	Fwd, CTGGGCATCGAGATCATCGG
	Rev, ATCCCACAGCCAGTGTAGTCA
HSP90B1	Fwd, CTGGGCATCGAGATCATCGG
	Rev, ATCCCACAGCCAGTGTAGTCA
